# Supplementary material for: Effects of nomadic grazing system and indoor concentrate feeding systems on performance, behavior, blood parameters, and meat quality of finishing lambs
Source: PLoS One. 2022 Dec 7;17(12):e0278669. doi: 10.1371/journal.pone.0278669 (PMC9728920; doi:10.1371/journal.pone.0278669)
Supplement: Attachment — Submitted filename: Comments to authors.docx [file pone.0278669.s002.docx]

**General comments:** This is an interesting study including many different parameters. The authors have chosen to present the results before materials and methods, however, I would prefer that the manuscript is arranged in the “traditional” way with materials and methods before the results. In my opinion, you need to know what has been done and in what way to be able to interpret the results in a good way. It is also a very high number of references (87), are all really necessary? In addition, the manuscript would benefit from being language edited.

| **Abstract**  General: Main objectives should be described in the abstract. No figures regarding welfare (behaviour) or feed efficiency are mentioned but you draw conclusions about these parameters as well. I would like to see a sentence covering these parameters. The whole abstract would benefit from being rewritten. As it is now you have only used 216 words out of the 300. | |
| --- | --- |
| L 15 | Write two instead of 2 |
| L 20-21 | Reformulate “Meat value at pH 0…” I miss information about pH 24 h which is more interesting than pH at 0 h from a eating quality perspective. |
| L 21-24 | The second sentence somewhat repeat what is already stated in the first sentence. Reformulate to avoid repetition. |
| L 24 | “…growth performance…” |
|  |  |
| **Introduction**  General: Also the introduction would benefit from being somewhat rewritten to get the text to flow. For example, the section L 41-58 have a lot of statements but do not really explain why e.g. meat quality is affected. And why is grass feeding discussed just because it is less efficient in growth rate? Here I would like to see pros and cons of both systems. Grazing can be less labour intensive, enhance biodiversity etc. | |
| L 33 | I can’t see that Wileman et al. state that consumers show an increasing interest in natural feeding regimes. Please find another source for that statement. With natural feeding, do you mean only grazing or feeding with roughages and cereals etc. without additives? |
| L 33-36 | These two sentences say similar things. Combine or omit one of them. |
| L 36-39 | Combine these two sentences. As it is written now it seems to be a contradiction between consumers’ interest in animal friendly production and high-quality food. Or do you mean just that? |
| L 41 | Delete “-“ between lambs and fed. |
| L 43-45 | Incomplete sentence. Feeding with concentrates do improve weight gain but is it really more profitable compared to grazing? The lambs are read for slaughter at an younger age but is also more expensive to feed concentrates vs. grazed grass. |
| L 52-54 | In what way do activity affect final pH? It is a huge difference between acute stress and exercise while grazing! |
| L 60 | You have not studied environmental impact nor profitability, or referred to papers stating this, hence you can’t state that the production systems studied effects these parameters. |
| L 61 | Don’t understand the sentence “Some parameters…”. |
| L 63 | Indoor rearing systems instead of indoor areas. |
|  |  |
| **Results**  General: You use many abbreviations that haven’t been explained before (they are explained in the materials and methods). I strongly advise you to move the material and methods section and place it before the results, but if you don’t do that you need to explain the abbreviations here. | |
| L 71-72 | Personally, I think feed composition is materials and methods rather than results since you have formulated the diets and they are a part of your experimental set up. Hence, I think you should move table 1 to the M&M section. Did you take any samples of the pasture? |
| Table 1 | I miss information about energy content of the diets. Correct unit for DM to g/100 g fresh weight under “Chemical composition” |
| L 72 | Explain the abbreviation FCR first time you mention it. |
| Table 2 | Add unit for FCR. Present hot carcass weight in kg instead of % and name the figures that you now present as HCW dressing instead, both numbers are interesting. |
| L 75 | “…final BW tended to be lower for NG lambs compared…” |
| L 77 | Further rather than however |
| L 79 | Growing season makes me think of plants and pasture, I would prefer rearing period or something equal. |
| L 80 | Fat tail weight was not higher in NG lambs |
| L 82-83 | NG lambs also spent more time drinking, could be worth mentioning. Did you measure time moving, walking? Would have been valuable since you state in the introduction that activity affect meat quality. |
| Table 5 | Review the parameters under the sub-heading Texture profile analysis, cooking loss, WHC and so forth isn’t really texture profile. |
| Figure 1 | I assume that the figures presented are accumulated over time? So it is not the changes between the months that is presented? Please clarify. |
| Figure 2 | This figure needs much more explanation to be of value for the reader. The figure (incl. figure text) should be able to explain it self, the reader should not need to read the text to understand. |
|  |  |
| **Discussion** |  |
| General: Sometimes it is not clear if you refer to your own results or to already published papers. Please, carefully review the discussion to avoid misunderstandings. | |
| L 120 | But you did not have any differences in eating or rumination time between MC and HC? Nor any differences in feed intake? So on what results do you base this statement? Or is this actually what you discuss? In that case, please clarify this part. |
| L 123-125 | This sentence is based on your own results, so why the reference? If the reference show similar results, clarify. |
| L 129 | Should it be concentrate:roughage ratio or the opposite? Please, clarify. |
| L 130-132 | The NG lambs did also gain weight and used energy for muscle development, please reformulate. And again, this is your results and not the results of Claffey et al. Separate your own results and results of others. Regarding profitability, you have not calculated on that. I suppose that you have a higher feed cost in the CON system, especially in the HC system and you raised the lambs equally long. Is the better performance of the CON lambs enough to get a better profit? From this study you can’t draw that conclusion. It can, however, be discussed and you can refer to others that might have shown a better profitability. |
| L 132-134 | Please, review and rewrite this sentence. |
| L 140 | Loin thickness |
| L 150-151 | Do you mean “…common in lambs fed grazed gras.”? |
| L 167 | Review this sentence. |
| L 173 | How do you know that feed intake was higher in NG lambs? They spent more time eating but that could be an indication of lower feed availability and not a higher feed intake per se. Most probably the indoor groups ate more. More drinking time could rather be an indication of a drier feed. How was the weather? Could it also be due to spending more time out in the sun (high temperature)? |
| L 195-196 | Review this sentence. |
| L 199 | Do you refer to your own study or to Alvarez-Rodriguez et al? It is not clear to me. Further, you did not measure rumen ammonia concentrations in your study. |
| L 201-203 | Review this sentence. |
| L 215-216 | You did not measure muscle glycogen content, hence cannot draw any conclusions based on that. |
| L 226 | I would not refer to feedlot as an extensive system. |
| L 233 | To understand what you mean with mixed group, one has to read the paper you refer to. Please clarify. |
| L 239-242 | Review these sentences. |
| L 243 | In what way was L* affected? Please specify. |
| L 247 | But you did not have any differences in chilling? |
| L 251-253 | The reference to French et al. can be deleted, or needs more clarification what you want to say with that. Sensory perception includes a variety of parameters, if it is just the perception of tenderness you want to highlight, specify. Further, you had differences in ADG between NG and CON so if you keep the reference, reformulate to also address your results. |
|  |  |
| **Conclusions** | |
| L 288 | But you did have significantly higher EBW and HCW in CON lambs compared to NG lambs. Reformulate. |
| L 292-293 | NG lambs gained weight, hence is was more than just maintenance (but a larger proportion of the energy intake was used for maintenance). I suppose you mead low-yielding and not high-yielding? |
| L 294 | You can’t say, from your study, that the CON lambs had a lower environmental impact. Delete that statement. |
|  |  |
| **Materials and methods** | |
| L 301-317 | In this section it is enough with stating that all treatments and procedures have been proven by the ethics committee. Other details about animal handling and slaughter procedures should be moved to the sections “Animals and experimental design” and “Slaughtering and sampling”. |
| L 320 | How old were the lambs at the start of the experiment? Were they weaned just prior to the experiment? |
| L 325 | What is your definition of nomadic pasture? Did you herd the lambs? Please specify that they were housed during night time. |
| L 327 | What did the NG-lambs eat while not on pasture? |
| L 329-330 | This sentence can be omitted, you say the same thing in the following sentence. |
| L 332 | Do you mean “…regimens during the 90-day…”? Delete (ii). |
| L 333 | Roughage:concentrate instead of feed:concentrate (concentrate is also a feed). Specify it is om DM basis (also for the HC group). |
| L 336 | One pen per group, I assume? How big were the pens? |
| L 341 | Whit this setup, with all animals in a group, you can’t assess individual feed intakes. It will only be a measure for the whole group and a mean per animal. |
| L 341-342 | How were the subsamples of feeds and feed residues taken? How samples stored before the subsamples were taken and how were the subsamples stored before analysis? |
| L 362 | Day 19 – is that 19 days after experimental start for the grazing lambs or is it 19 days after the indoor groups started on their respective treatments? Or were the blood samples not taken on the same day for all groups? Why did you not take blood samples later during the experiment? |
| L 387-389 | Check the language. |
| L 390 | Define what you include in GIT (RR is also a part of the whole GIT) |
| L 401-403 | Did you measure colour on the surface of the LD while still intact on the carcasses? Did you dress that muscle separately from fat? Usually, colour is measured after letting the muscle bloom for some time. |
| L 404 | Delete ±, gets confusing |
| L 418 | The sentence needs to be rewritten. Did you keep the samples vacuum packed between the drip loss measurements? Or how were the samples stored? And please find another reference for the drip loss. There are several papers dealing with this which are much more suitable than this one about shrimps. |
|  |  |
|  |  |
|  |  |
