# Supplementary material for: Effects of nomadic grazing system and indoor concentrate feeding systems on performance, behavior, blood parameters, and meat quality of finishing lambs
Source: PLoS One. 2022 Dec 7;17(12):e0278669. doi: 10.1371/journal.pone.0278669 (PMC9728920; doi:10.1371/journal.pone.0278669)
Supplement: Attachment — Submitted filename: Response to Reviewers.docx [file pone.0278669.s003.docx]

PLOS ONE

**PONE-D-21-28984**

**Effects of nomadic-grazing and indoor concentrate feeding systems on growth performance, behavior, blood parameters, and carcass quality of finishing lambs**

**Journal Requirements:**

When submitting your revision, we need you to address these additional requirements.

1. Please ensure that your manuscript meets PLOS ONE's style requirements, including those for file naming. The PLOS ONE style templates can be found at 
<https://journals.plos.org/plosone/s/file?id=wjVg/PLOSOne_formatting_sample_main_body.pdf> and 
<https://journals.plos.org/plosone/s/file?id=ba62/PLOSOne_formatting_sample_title_authors_affiliations.pdf>

AU: The manuscript has been revised to meet the style requirements of PLOS ONE.

2. We suggest you thoroughly copyedit your manuscript for language usage, spelling, and grammar. If you do not know anyone who can help you do this, you may wish to consider employing a professional scientific editing service.  Whilst you may use any professional scientific editing service of your choice, PLOS has partnered with both American Journal Experts (AJE) and Editage to provide discounted services to PLOS authors. Both organizations have experience helping authors meet PLOS guidelines and can provide language editing, translation, manuscript formatting, and figure formatting to ensure your manuscript meets our submission guidelines. To take advantage of our partnership with AJE, visit the AJE website (<http://aje.com/go/plos>) for a 15% discount off AJE services. To take advantage of our partnership with Editage, visit the Editage website ([www.editage.com](http://www.editage.com)) and enter referral code PLOSEDIT for a 15% discount off Editage services.  If the PLOS editorial team finds any language issues in text that either AJE or Editage has edited, the service provider will re-edit the text for free. Upon resubmission, please provide the following:
 
The name of the colleague or the details of the professional service that edited your manuscript

A copy of your manuscript showing your changes by either highlighting them or using track changes (uploaded as a *supporting information* file)

A clean copy of the edited manuscript (uploaded as the new *manuscript* file)”"

AU: We revised the manuscript based on the advice and suggestions provided by Editage's Scientific Editing Service (https://www.editage.com/quality), which specializes in scientific and academic language editing. The revised version was submitted with an editing certificate (Requested under: INQ_IMARH_1) as well.

AU: We revised the manuscript based on the advice and suggestions provided by Editage's Scientific Editing Service (https://www.editage.com/quality), which specializes in scientific and academic language editing. The revised version was submitted with an editing certificate (Requested under: INQ_IMARH_1) as well a copy of your manuscript showing your changes by either highlighting them or using track changes.

3. Thank you for stating the following in the Acknowledgments Section of your manuscript:

The authors gratefully acknowledge Isfahan University of Technology (Isfahan, Iran) and the Nomadic Affairs Department for the financial support and research services extended to the authors.

We note that you have provided additional information within the Acknowledgements Section that is not currently declared in your Funding Statement. Please note that funding information should not appear in the Acknowledgments section or other areas of your manuscript. We will only publish funding information present in the Funding Statement section of the online submission form. 
Please remove any funding-related text from the manuscript and let us know how you would like to update your Funding Statement. Currently, your Funding Statement reads as follows:

The author(s) received no specific funding for this work.

Please include your amended statements within your cover letter; we will change the online submission form on your behalf.

AU: We have revised the Acknowledgements Section in order to remove the funding statement. The Funding Statement does not need to be updated.

4. We note that you have stated that you will provide repository information for your data at acceptance. Should your manuscript be accepted for publication, we will hold it until you provide the relevant accession numbers or DOIs necessary to access your data. If you wish to make changes to your Data Availability statement, please describe these changes in your cover letter and we will update your Data Availability statement to reflect the information you provide.

AU: We prefer not to provide repository information at the acceptance of our data. For this reason, we would like to make changes to our data availability statement. These changes have been described in our cover letter, and our data availability statement has been updated to reflect the new information.

**Academic Editor comments:**

The manuscript was reviewed by experts and requires major revision before it can be considered for publication. Please take into consideration the points raised by reviewers, especially reviewer#1 when revising your manuscript.

**Response to Academic Editor:**

Dear Dr. Antonio Humberto Hamad Minervino,

AU: We appreciate the opportunity to revise and resubmit this manuscript. Thank you for your constructive feedback on our original submission. We greatly appreciate the comments and criticisms. We revised the manuscript based on the advice and suggestions provided by Editage's Scientific Editing Service (https://www.editage.com/quality), which specializes in scientific and academic language editing. The revised version was submitted with an editing certificate (Requested under: INQ_IMARH_1) as well. We have addressed the reviewers' comments and questions in detail below and have made the necessary changes to the text. The changes and additions are highlighted in yellow in the revised manuscript.

Sincerely,

Morteza H. Ghaffari

However, I would prefer that the manuscript is arranged in the “traditional” way with materials and methods before the results. In my opinion, you need to know what has been done and in what way to be able to interpret the results in a good way. In addition, the manuscript would benefit from being language edited.

*AU: The manuscript was revised based on your guidance on materials and methods before the results and manuscript were provided by Editage's Scientific Editing Service (https://www.editage.com/quality), which specializes in technical and academic language editing.*

Main objectives should be described in the abstract. No figures regarding welfare (behaviour) or feed efficiency are mentioned but you draw conclusions about these parameters as well. I would like to see a sentence covering these parameters. The whole abstract would benefit from being rewritten. As it is now you have only used 216 words out of the 300.

*AU: Thank you for your constructive comments. We have revised the Abstract section to reflect the findings according to your suggestions.*

pH 24 h which is more interesting than pH at 0 h from an eating quality perspective.

*AU: We have revised the Abstract section to reflect the findings according to your suggestions (Lines 24-25).*

The second sentence somewhat repeat what is already stated in the first sentence. Reformulate to avoid repetition.

*AU: Revisions were made to prevent repetition.*

Also the introduction would benefit from being somewhat rewritten to get the text to flow. For example, the section L 41-58 have a lot of statements but do not really explain why e.g. meat quality is affected. And why is grass feeding discussed just because it is less efficient in growth rate? Here I would like to see pros and cons of both systems. Grazing can be less labour intensive, enhance biodiversity etc.

*AU: Thank you for your suggestion. We have revised the Introduction section to reflect the findings according to your suggestions “Considering that feed costs often represent a significant portion of the total variable costs of lamb production, this favorable environment for abundant grass growth results in pasture grass being the most economical feed source [1]. In addition, meat from lambs raised on pasture contains less subcutaneous backfat than meat from lambs raised in pens, which is more attractive to consumers [2]. Therefore, consumers are becoming increasingly interested in sustainable and animal-friendly production and a healthy diet [3].*

*There has been much discussion about the rearing of grass-fed lambs, as lambs fed concentrates are more efficient than lambs raised on pasture [4]. The improvement of energy supply by concentrates offered to lambs in the barn increases weight gain and eventually carcass yield [5]. In addition, consumption of starch concentrates increases propionate content in the rumen, which stimulates insulin secretion and fat synthesis [6]. The intensity of the production system, as measured by daily growth rate (daily live weight gain), has been shown to have significant effects on meat quality, particularly composition (% lipid) and color [7]. Meat quality can be affected by exercise, especially its color [8]. Previous studies have shown that exercise before slaughter increases the tenderness of the longissimus muscle and drip loss of lambs [9]. Cows that are constantly grazed or kept in an extensive environment have darker muscle color [1].” (Lines 40-55).*

I can’t see that Wileman et al. state that consumers show an increasing interest in natural feeding regimes. Please find another source for that statement. With natural feeding, do you mean only grazing or feeding with roughages and cereals etc. without additives?

*AU: Thank you for your constructive comments. We have revised the Introduction section to reflect the findings according to your suggestions (Lines 40-42).*

You have not studied environmental impact nor profitability, or referred to papers stating this, hence you can’t state that the production systems studied effects these parameters.

*AU: Thank you for your constructive comment. Based on your suggestions, we revised the sentence and removed environmental impact and profitability.*

You use many abbreviations that haven’t been explained before (they are explained in the materials and methods). I strongly advise you to move the material and methods section and place it before the results, but if you don’t do that you need to explain the abbreviations here.

*AU: Your comment has been considered and the text has been modified for abbreviations* *accordingly. The material and methods section have been moved before the results section.*

Personally, I think feed composition is materials and methods rather than results since you have formulated the diets and they are a part of your experimental set up. Hence, I think you should move table 1 to the M&M section. Did you take any samples of the pasture?

*AU: Thank you for your constructive comment .Table 1 has been moved to Materials & Methods. Despite taking samples, no analysis has been conducted on the pasture samples.*

I miss information about energy content of the diets. Correct unit for DM to g/100 g fresh weight under “Chemical composition” and Add unit for FCR

*AU: Thanks for your comment. Metabolizable energy (Mcal/kg DM) of the diets added in Table 1. In response to your question, chemical composition, expressed in terms of dry matter not fresh weight. To determine DM and nutrient composition, representative samples of forages, treatment TMR, and individual refusals were collected immediately before the morning feeding during the 7-d collection period. All samples were frozen immediately at −20 °C until analyses.* *After thawing, Weekly feed and ort samples were treated at 60 °C in a forced-air oven (Memmert, Schwabach, Germany) for 48 h and then ground to a size less than 1 mm using a Wiley mill (Arthur H. Thomas; Philadelphia, PA, USA). Samples were analyzed in triplicate according to Association of Official Agricultural Chemists (AOAC) [11] for crude protein (CP) by the Kjeldahl method (Kjeltec 1030 Auto Analyzer, Tecator, Höganäs, Sweden; method 955.04), ash (method 942.05), ether extract (method 920.39), and neutral detergent fiber (NDF) using an ANKOM200 Fiber Analyzer (A200 model, Ankom Technology, Macedon, NY) according to the manufacturer's recommended reagents and filter bags (#F57) with pore size of 25 μm. NDF content was analyzed with a heat-stable α-amylase and without sodium sulfite, and was expressed as acid-detergent fiber without considering residual ash. Ash content was measured after combustion in a muffle furnace at 550 °C overnight. The content of non-structural carbohydrates in the feed was calculated on a dry matter basis [10] as follows: 100− (NDF%+CP%+ether extract%+ash%). However, there is no unit associated with FCR.*

Present hot carcass weight in kg instead of % and name the figures that you now present as HCW dressing instead, both numbers are interesting.

*AU: Thank you for your comment. Hot carcass weight as percentage of carcass weight at slaughter and kg reported on Table 2.*

NG lambs also spent more time drinking, could be worth mentioning. Did you measure time moving, walking? Would have been valuable since you state in the introduction that activity affect meat quality.

*AU: Thanks for your questions. We were unable to measure walking time in NG lambs due to the difficulty of the measurement.*

Figure 1: I assume that the figures presented are accumulated over time? So it is not the changes between the months that is presented? Please clarify.

*AU: Thanks for the question. However, drip loss and TBARS level did not differ between the meat of NG and CON-fed animals; instead, as expected, drip loss and lipid oxidation (P<0.01) increased with the duration of storage (Fig 1A–B).*

This figure needs much more explanation to be of value for the reader. The figure (incl. figure text) should be able to explain it self, the reader should not need to read the text to understand.

*AU: In the spirit of your suggestion, the legends of Figures 1 and 2 have been revised to make them self-explanatory.*

But you did not have any differences in eating or rumination time between MC and HC? Nor any differences in feed intake? So on what results do you base this statement? Or is this actually what you discuss? In that case, please clarify this part.

*AU: We revised the manuscript based on your suggestions and deleted this part from discussion.*

The NG lambs did also gain weight and used energy for muscle development, please reformulate.

*AU: Thank you for your comment. The EBW and HCW were influenced by finishing system (P<0.05) and were higher in CON-fed lambs than in NG lambs. This difference in HCW is probably due to high energy intake and consequent high growth rate, which from a commercial point of view emphasizes the importance of finishing diets with low indigestible fiber content to achieve maximum live weight gains and ultimately high carcass weights (Lines 376-380).*

How do you know that feed intake was higher in NG lambs? They spent more time eating but that could be an indication of lower feed availability and not a higher feed intake per se. Most probably the indoor groups ate more. More drinking time could rather be an indication of a drier feed. How was the weather? Could it also be due to spending more time out in the sun (high temperature)?

*AU: Thank you for your constructive comments. We have revised the conclusions section to reflect the findings according to your suggestions. In this study, drinking and standing times were higher (P<0.01), but resting time was lower (P<0.01) in NG lambs than in CON-fed lambs, which was related to finding food for eating and more standing time. It is likely that the indoor groups ate more than the outdoor groups. It is possible that the longer eating time is an indication of lower feed availability rather than higher feed intake per second. In addition, the longer drinking time could be an indication of a drier diet (Lines 434-439).*

I would not refer to feedlot as an extensive system.

*AU: Thank you for your constructive comment. The feedlot deleted as an extensive system in paragraph.*

In what way was L* affected? Please specify.

*AU: Thanks for your question. Meat color is influenced by post mortem factors basically involve the refrigerated storage of meat, often termed aging [71]. Protein denaturation during rigor mortis increased free water and juice loss, resulting in changes in light scattering [72]. Therefore, the increase in L* during storage is consistent with the results obtained in chilled beef, suggesting that the difference in L* 24 h post mortem could be partially due to the differences in chilling [73].*

But you did not have any differences in chilling?

*AU: Thanks for your question. However, drip loss and TBARS level did not differ between the meat of NG and CON-fed animals; instead, as expected, drip loss and lipid oxidation (P<0.01) increased with the duration of storage (Fig 1A–B).*

What is your definition of nomadic pasture? Did you herd the lambs? Please specify that they were housed during night time.

*AU: Thank you for your constructive questions. The lambs were randomly assigned to three production systems: NG (n =10), the where animals were housed in loose pens and raised on green nomadic pasture (nomadic livestock breeding on rangelands has been common place as a source of livelihood in different parts of Iran approximately 150 ha) for 14 h per day (0500–1800 h) and they were housed during night time until slaughter. The grazing season lasted 90 days (February 1, 2019 to May 1, 2019). (Lines 82-86).*

One pen per group, I assume? How big were the pens? Whit this setup, with all animals in a group, you can’t assess individual feed intakes. It will only be a measure for the whole group and a mean per animal.

*AU: Thank you for your questions. The lambs that were handled indoors were housed in a single pen (1.2 m × 1.5 m) (Lines 91-92).*

How were the subsamples of feeds and feed residues taken? How samples stored before the subsamples were taken and how were the subsamples stored before analysis?

*AU: Thank you for your constructive questions. Samples of forages, subsamples of feeds, and individual refusals were collected immediately before the morning feeding during the 7-d collection period and placed into square nylon. All samples were frozen immediately at −20 °C until analyses. After thawing, weekly feed and ort samples were treated at 60 °C in a forced-air oven (Memmert, Schwabach, Germany) for 48 h and then ground to a size less than 1 mm using a Wiley mill (Arthur H. Thomas; Philadelphia, PA, USA) for each individual lamb (Lines 106-111).*

Day 19 – is that 19 days after experimental start for the grazing lambs or is it 19 days after the indoor groups started on their respective treatments? Or were the blood samples not taken on the same day for all groups? Why did you not take blood samples later during the experiment?

*AU: In response to your question, blood samples were collected on d 19 after experimental start grazing lambs and indoor groups started by venipuncture of the coccygeal vein (10 mL serum clot activator evacuated tubes) 4 h post-feeding from all lambs as a same day.time (Line 132-134). I did not take blood samples later during the experiment because its many expensive and our financial budget was limited.*

Did you measure color on the surface of the LD while still intact on the carcasses? Did you dress that muscle separately from fat? Usually, color is measured after letting the muscle bloom for some time

*AU: Thank you for your constructive questions. The meat color of LD muscle at the thirteenth thoracic vertebra was rested 4 h at approximately 7 ^o^C and was then cut into slices, each with a thickness of 2.5 cm (first measurement) and 24 h post mortem (second measurement) (Lines 176-178).*

The sentence needs to be rewritten. Did you keep the samples vacuum packed between the drip loss measurements? Or how were the samples stored? And please find another reference for the drip loss. There are several papers dealing with this which are much more suitable than this one about shrimps.

*AU: Thank you for your questions. I find another reference for the drip loss:*

*Honikel KOJMs. Reference methods for the assessment of physical characteristics of meat. Meat Sci, 1998;49(4):447-57.* [*https://doi.org/10.1016/S0309-1740(98)00034-5*](https://doi.org/10.1016/S0309-1740(98)00034-5)*.*

*In response to your question, meat samples are cut from the carcass and immediately weighed. A sample weight of approximately 80-100g is recommended. The samples are placed within the container on the supporting mesh and sealed. After a storage period (usually 24 h) at chill temperatures (1 to YC), samples are again weighed. The same samples can be used for further drip loss measurements, e.g. after 1,2,3, 4 months, etc., but in every case the initial weight is used as the reference point. At the time of measurement, samples should be taken immediately from the containers, gently blotted dry and weighed [15] (Lines 190-197).*

**Response to Reviewer 1 Comments:**

The conclusion needs to be partly rewritten to reflect the results and to not draw conclusions based on assumptions.

*AU: Thank you for your constructive comments. We have revised the conclusions section to reflect the findings according to your suggestions. I hope the current draft is clearer and more focused on the research objective associated with the results.*

Further, the manuscript would benefit from being language edited, both due to grammatical issues as well as incomplete, or wrongfully, sentences.

*AU: We revised the manuscript based on the advice and suggestions provided by Editage's Scientific Editing Service (https://www.editage.com/quality), which specializes in scientific and academic language editing. The revised version was submitted with an editing certificate (Requested under: INQ_IMARH_1) as well.*

**Response to Reviewer 2 Comments:**

Abstract does not have a suitable structure. In the abstract, after stating the challenges and objectives of the research, the main points of the methodology should be stated and then the results, discussion and general conclusion should be stated.

*AU: Thank you for your constructive comment. We have revised the Abstract section according to your suggestions.*

I did not understand how the lambs were slaughtered. Has the HALAL method been performed or not?

*AU: Yes. Halal method was used. This information has been added in the manuscript.*

Authors state that they have allowed 20% of the feed left in the bunk. This method allows the lambs to sort the diet and change their feeding behavior. Are the nutrients in the ort measured to calculate the amount of nutrients consumed?

*AU: Yes, they were measured. A subsample of the uneaten feed residues was collected once a week for calculation of nutrient intake.*

Significant differences in RR and GIT weights between treatments were not correctly interpreted. The mass of these parts is a function of the supply of energy and protein to the animal, which is higher with concentrate diets than with forage diets.

*AU: Your comment has been considered and the text has been modified accordingly as follows: The rumen passage rate of legumes is usually higher than that of grasses because legumes pass through the rumen easily due to particle breakdown [26]. This may be attributed to the fractional rumen passage rate, as grasses may decrease the fractional rumen passage rate and increase the amount of undigested feed reaching the abomasum, which could increase the weight of abomasum tissue [27]. It is important to note that under natural rearing conditions, especially when young ruminants are kept on pasture, most of the nutrients and stimulants for rumen development are obtained from fresh forage. Large particles, fresh forage, and high fiber sources provide physical stimuli that increase rumen motility, muscularization, and rumen volume in calves [28]. Physical stimulation through forage intake increases rumen weight [29]. In fact, RR masses increase due to an increase in RR weight and muscle development [30].*

Ruminating time is longer with concentrate diets. This result may be a function of the sorting of the given diet or the animal's adaptation mechanisms to concentrate diets. A more appropriate interpretation is needed. It is also recommended that feeding behaviors be stated and interpreted as min to dry matter and fiber intake.

*AU: Thank you for your constructive comment. We fully agree with your comment and the part of this result was not expected. The text has been modified accordingly (Lines 418-434).*
