# Supplementary material for: Effects of nomadic grazing system and indoor concentrate feeding systems on performance, behavior, blood parameters, and meat quality of finishing lambs
Source: PLoS One. 2022 Dec 7;17(12):e0278669. doi: 10.1371/journal.pone.0278669 (PMC9728920; doi:10.1371/journal.pone.0278669)
Supplement: Attachment — Submitted filename: Comments to authors rev 1.docx [file pone.0278669.s004.docx]

| **General comments** | |
| --- | --- |
| The manuscript have improved but there are still some aspects that need to be dealt with before it is suitable for publication. Even though the manuscript has been language edited there still are sentences, or sections, that needs to be rewritten. I have found some of the references that are wrongly used. In addition, you still have a very large number of references and by just reading the title of them I doubt that all of them are correctly used or necessary for this manuscript. Be really careful that you cite the papers correct!  In some cases, it is still confusing if you in the discussion refer to your own study or to other papers. Please, be clear about that. | |
|  |  |
| **Abstract** |  |
| L 22-23 | …average age of 90 ± 4 days (mean ± SD)… And the same for 180 days. |
| L 24 | You haven’t explained CON, write out in full first time you mention it. |
| L 25 | What do you mean with “meat values”? If you refer to pH in the meat write instead “higher pH values”. |
| L 26 | I assume you mean “24” and not “4”? |
| L 35 | The fact that MC lambs ate less concentrate is not a result since you decided how much they were offered. Please delete that part. |
|  |  |
| **Introduction** | |
| L 44-45 | This sentence could be placed first as a statement. |
| L 49-50 | Since you state that consumers prefer less fat, this would be a draw back with concentrate feeding? |
| L 52 | If you refer only to fat content it is better to write that rather than “composition”. |
| L 53-54 | Reference no. 9 did not see any differences neither in WBSF nor in drip loss due to exercise stress. They just mention that others have seen that. Replace the reference or omit the sentence. |
| L 77-78 | Place 90 and 180 outside the parenthesis. |
| L 83 | …the where animals were… Check the sentence and rephrase. |
| L 83-84 | Within parenthesis, I assume you want to say that the pasture you used was 150 ha, the rest of the sentence can be omitted. |
| L 91-92 | “Reared” instead of “handled”. I assume you housed one lamb in each pen but as the sentence is now one can interpret it as you housed all lambs in one single pen, please rephrase. |
| L 93-94 | It is unclear to me what you mean with this sentence. You refer to NRC requirements for dairy cattle and not to a study where the diets are presented. |
| L 95 | The lambs did not refuse feed intake, they left 20% refusals of feed offered. Rephrase the sentence. |
| L 97 | Did you only take samples of the orts to analyse nutrient composition? Lambs are good at sorting the feed so by only analysing the orts you will not get a picture of what the lambs actually have eaten. Or do you mean that you calculated the feed intake? Did you feed forages and concentrates separately or as TMR? Please clarify. |
| L 107 | Square nylon? Please specify what you mean. |
| L 121 | Just some “left overs” from earlier versions, I assume. |
| Table 1 | Yes, I understand that the chemical composition is presented as g/100 g DM but DM itself is not g/100 g DM (then it would be 100% in your table), rather g/100 g fresh weight. All other components are in g/100 g DM, as you correctly have stated. |
| L 132-133 | Please, rephrase the sentence. |
| L 153 | Either delete NG or rephrase. |
| L 153-154 | Move this sentence to “Animals and experimental design” |
| L 158-159 | You slaughtered all animals the same day (180) and not by a target weight, please rephrase. |
| L 165-166 | Review the sentence. |
| L 174 | On line 168 you state that you took a sample from the right half of the carcasses for meat quality measurements but here you state that you measured on the left half. Which is correct? |
| L 176-178 | How you conducted the colour measurements is still confusing to me. Did you cut out the LD muscle right after slaughter and sliced it? Did you measure on the same piece of meat all the time or on different slices? Or did you measure on several slices at each time? Why did you do it immediately after slaughter? The most common is to do it after chilling and cutting the carcass. Did you proceed with the other meat quality measurements on the same sample? Cutting out the LD at such an early stage will effect e.g tenderness. |
| L 184 | The sample was then weighed. Should this sentence be there? |
| L 190-197 | This part is directly taken from the book “Handbook of Muscle Foods Analysis”. It is not ok to take a text verbatim from another source! |
| L 205-212 | WHC is a property of raw meat that describes the ability to bind water. It can be measured in different ways, e.g. as drip loss. When you heat the meat it is rather cooking loss you get. I can’t see that the method you describe is presented in the paper of Graham Trout. Why centrifuge at 10,000 g, when this is usually done at ordinary gravity (1 g) when it comes to drip loss? |
| L 277-278 | You have no values of feed intake or FCR for NG. Reformulate to levels of concentrate instead of feeding indoors. |
| L 281-282 | Loin thickness did only differ between NG and CON, not between MC and HC. Please specify. |
| L 284, Table 2 | RR is a part of the GIT, as you now have defined it in M&M, and should be presented as such also here. |
| Table 3 | It is enough of you present the result in whole minutes. Since you assumed that the behaviour continued for 5 min you don’t have that precision to actually measure in seconds. |
| L 322 | …indicated that chemical composition of the meat did not differ… |
| L 330 | Delete “…in the CON fed lamb meat than that in NG lamb meat.” |
| Table 5 | Cooking loss, is it really in g/kg? WHC, what does the figures really mean? As said before, WHC is usually measured as drip loss so the latter would have been more proper to present in the table. |
|  |  |
| **Discussion** |  |
| L 371-373 | These two sentences say almost the same (ADG= weight gain). |
| L 384 | Again, RR is a part of GIT. |
| L 400 | I assume you mean low fat cover for NG lambs? However, in your study you could not see a difference in back fat cover among the treatments. Or do you refer to another study? This is also valid for L 401-403, you own or someone else’s study? |
| L 414 | Rephrase “…the consume energy and energy cost…” |
| L 418 | “Ruminating” instead of “rumination”. |
| L 426-427 | This sentence do not contribute to the text and can be omitted. |
| L 437 | There was only one outdoor group. |
| L 437-438 | I think it is quite obvious that longer eating time is due to lower feed availability. Review if you really need the long reasoning above to come to that conclusion. |
| L 441-450 | But is longer eating time always a bad thing, is the welfare worse? Of course, if the animals are starving but their natural behaviour is to seek for feed a large part of the day. I miss a discussion about “optimal” eating time, which automatically does not lead to a lower welfare due to longer eating time. |
| L 480 | Meat quality |
| L 497 | “…in the mixed group”, you need to explain what this means. |
| L 504 | Rephrase “…, that aging improved…”. |
| L 505-510 | But did you have differences in chilling between the treatments? If so, this is confounding and should have been stated in the M&M. If you did not have different chilling, why do you think this could be the reason? |
| L 516 | Pre-slaughter |
| L 518 | Is 75 really the right reference? Earlier you referred to another paper when writing about WHC. And once again, WHC is a property of the raw meat and drip loss is used to describe it. Cooking loss is cooking loss and nothing else. |
|  |  |
| **Conclusion** |  |
| L 549 | To state that the NG lambs had lower welfare just due to longer eating time is doubtful. Then you need to put that in relation to what a “normal” eating time is. I think you should delete this statement. |
| L 551 | You haven’t discussed profitability at all in the discussion. I agree that they probably are since they ate less concentrate but performed as well as HC. However, to state this I think you should mentioned this in the discussion as well. |
