# Supplementary material for: Effects of nomadic grazing system and indoor concentrate feeding systems on performance, behavior, blood parameters, and meat quality of finishing lambs
Source: PLoS One. 2022 Dec 7;17(12):e0278669. doi: 10.1371/journal.pone.0278669 (PMC9728920; doi:10.1371/journal.pone.0278669)
Supplement: Attachment — Submitted filename: Response to Reviewers.docx [file pone.0278669.s005.docx]

ACADEMIC EDITOR: The manuscript was improved but there are still major issues that need to be addressed before it can be considered for publication. Please take into consideration the comments made by the reviewer when revising the manuscript. In addition, I believe that a figure that illustrates the three different feeding systems would improve the manuscript. You can this new figure in the methods section. Pay special attention in the references, as mentioned by the reviewer.

*AU: Thank you for your comments. The manuscript has been revised based on your comments. The section on materials and method has been updated with illustrations showing different feeding systems. In addition, some of the references have been deleted or changed.*

Another suggestion: The table 3 could be transformed into a stacked column graph, providing a better visual representation of your data.

*AU: Thank you for your suggestion. We transferred table 3 to column graph to better visual representation of our data.*

**General comments**

The manuscript have improved but there are still some aspects that need to be dealt with before it is suitable for publication. Even though the manuscript has been language edited there still are sentences, or sections, that needs to be rewritten. I have found some of the references that are wrongly used. In addition, you still have a very large number of references and by just reading the title of them I doubt that all of them are correctly used or necessary for this manuscript. Be really careful that you cite the papers correct!

In some cases, it is still confusing if you in the discussion refer to your own study or to other papers. Please, be clear about that.

*AU: Thank you for your comments. The sentences have been reworded as much as possible. The number of references have been reduced. We have reviewed all references and deleted some; others have been corrected or revised based on citations in the paper. In writing, we tried to eliminate doubts and change or delete them when they were not needed in the sentences.*

**Abstract**

…average age of 90 ± 4 days (mean ± SD) and the same for 180 days.

*AU: Thank you for your comments. According to your suggestions, was corrected.*

You haven’t explained CON, write out in full first time you mention it.

*AU: Thank you for your constructive comments. We have revised this section according to your suggestions.*

What do you mean with “meat values”? If you refer to pH in the meat write instead “higher pH values”.

*AU: Thank you for your suggestion, we have revised the sentence.*

I assume you mean “24” and not “4”?

*AU: Thank you for your comment. Yes, the 24 is right and it was corrected.*

The f act that MC lambs ate less concentrate is not a result since you decided how much they were offered. Please delete that part.

*AU: Thank you for your constructive comments. We have removed that part and corrected.*

**Introduction**

This sentence could be placed first as a statement.

*AU: Your comment has been considered and the sentence have been moved first as a statement (Lines 39-40).*

Since you state that consumers prefer less fat, this would be a draw back with concentrate feeding?

*AU: Thank you for your comment. Yes, you are right. Meat from lambs raised on pasture contains less subcutaneous backfat than meat from lambs raised in pens, which is more attractive to consumers [2]. Diaz et al [2] reported that in sheepfold lamb dorsal fat thickness, kidney knob and cannel fat, and percent leg fat were higher in lambs raised in pens than in lambs raised on pasture. Lambs with higher weight had more fat. Considering that feed costs often represent a significant portion of the total variable costs of lamb production, this favorable environment for abundant grass growth results in pasture grass being the most economical feed source. In addition, pasture grass has a high content of natural antioxidants and therefore provides animals with better protection against lipid oxidation [3]. According to Santé-Lhoutellier et al [4], the oxidative stability of lamb is related to its diet. Pasture feeding offers some advantages over concentrate feeding in terms of lipid oxidation and, to a lesser extent, protein oxidation [4].*

*There has been much discussion about raising grass-fed lambs because lambs fed concentrates are more efficient than lambs raised on pasture [5]. By giving concentrates to lambs in the barn, it is possible to improve weight gain, carcass yield, and ultimately the profitability of the production system [6]. Color of meat is also an important factor in consumer selection. In particular, exercise can influence color and flavor [7]. It has been shown that cows that are constantly on pasture or kept in an extensive environment have a darker muscle color [8] (Lines 40-56).*

If you refer only to fat content it is better to write that rather than “composition”.

*AU:* *Thank you for your comment and your comment was used.*

Reference no. 9 did not see any differences neither in WBSF nor in drip loss due to exercise stress. They just mention that others have seen that. Replace the reference or omit the sentence.

*AU: Thank you for your* *detailed examination. The reference no. 9 was deleted.*

Place 90 and 180 outside the parenthesis.

*AU: Your comment has been considered and we place 90 and 180 outside the parenthesis.*

…the where animals were… Check the sentence and rephrase.

*AU:* *Thank you very much for your comment. The sentence has been rewritten.*

Within parenthesis, I assume you want to say that the pasture you used was 150 ha, the rest of the sentence can be omitted.

*AU: Thanks for your explanation and the sentence was shortened.*

“Reared” instead of “handled”. I assume you housed one lamb in each pen but as the sentence is now one can interpret it as you housed all lambs in one single pen, please rephrase.

*AU: Thank you for your constructive comment and we used* “*Reared” instead of “handled*”.

It is unclear to me what you mean with this sentence. You refer to NRC requirements for dairy cattle and not to a study where the diets are *presented.*

*AU: Thank you very much for your comment. The reference was corrected and we used the NRC (2007) requirements for lamb (reference no. 9).*

The lambs did not refuse feed intake, they left 20% refusals of feed offered. Rephrase the sentence.

*AU: Your comment has been considered and rephrase the sentence based on your comment (Lines 99-100).*

Did you only take samples of the orts to analyse nutrient composition? Lambs are good at sorting the feed so by only analysing the orts you will not get a picture of what the lambs actually have eaten. Or do you mean that you calculated the feed intake? Did you feed forages and concentrates separately or as TMR? Please clarify.

*AU: Thanks for your questions. The sentence was rewritten. Each morning, the entire backyard was removed and the feed consumption was then measured. Representative samples were taken from each stall for further analysis (Lines 100-102). We feed forages and concentrates as TMR.*

Square nylon? Please specify what you mean.

*AU: Thank you for your attention. I mean nylon bag stock and it has been corrected.*

Just some “left overs” from earlier versions, I assume.

*AU: Thank you for your careful look and yours was right. The sentence was deleted.*

Yes, I understand that the chemical composition is presented as g/100 g DM but DM itself is not g/100 g DM (then it would be 100% in your table), rather g/100 g fresh weight. All other components are in g/100 g DM, as you correctly have stated.

*AU:* *Thank you for your comment. Yes, your comment is right and it was corrected and Dry matter is % of fresh fed weight. It has been clarified in Table 1.*

Please, rephrase the sentence.

*AU: Thank you for your comment. The sentence was rephrased.*

Either delete NG or rephrase.

*AU: Thank you for your comment and NG has been deleted.*

Move this sentence to “Animals and experimental design”.

*AU: Thank you for your suggestion and this sentence has been moved to “Animals and experimental design”.*

You slaughtered all animals the same day (180) and not by a target weight, please rephrase.

*AU: Thank you for your constructive comment. The sentence has been rephrased.*

Review the sentence.

*AU: Thank you for your comment. The sentence was review and deleted because it not necessary.*

On line 168 you state that you took a sample from the right half of the carcasses for meat quality measurements but here you state that you measured on the left half. Which is correct?

*AU:* *Thank you for your careful review and your question. Right half is correct and the sentence has been corrected.*

How you conducted the color measurements is still confusing to me. Did you cut out the LD muscle right after slaughter and sliced it? Did you measure on the same piece of meat all the time or on different slices? Or did you measure on several slices at each time? Why did you do it immediately after slaughter? The most common is to do it after chilling and cutting the carcass. Did you proceed with the other meat quality measurements on the same sample? Cutting out the LD at such an early stage will effect e.g tenderness.

*AU:* *Thank you for your questions. I'm sorry to confusing you.*

*Answer to question 1: Yes, LD muscles are cut out and sliced immediately after slaughter.*

*Answer to question 2: The different pieces of meat (muscle LD) we used for the measurement. However, the color of the meat was obtained from the thirteenth thoracic vertebra immediately after slaughter and at 24 hours post mortem in the same piece.*

*Answer to question 3: The color of meat depends on the concentration of a haeminic pigment, myoglobin, and its oxidation state (Renerre, 1990). Color measurements were taken immediately after slaughter and 24 hours postmortem to determine the effects of storage time (Sen et al., 2011) and color stability.*

*Answer to question 4: The other measurements were made on the longissimus dorsi muscle, but I used the same sample to measure color 0- and 24-hours postmortem.*

*Answer to question 5: Longissimus dorsi muscle was excised from the right half carcass and sampled for the following meat quality determinations immediately after slaughter (Lines 167-169). The color of a meat cut (2.5 cm-thick) from the thirteenth thoracic vertebra, was determined at 0 h post mortem (immediately after slaughter) and 24 h post mortem in the same section (Lines 175-177).*

*Response to Question 6: We agree with the reviewer's position. Removal of the LD at an early stage may result in tenderness, and we can take that into consideration. However, the objective was to compare the 3 treatments and, the effect of cutting at an early stage was identical for all of them.*

The sample was then weighed. Should this sentence be there?

*AU: Thanks for your comment. This sentence has been deleted.*

This part is directly taken from the book “Handbook of Muscle Foods Analysis”. It is not ok to take a text verbatim from another source!

*AU: Thank you for your comment. An edited sentence replaced the previous one (Lines 188-193).*

WHC is a property of raw meat that describes the ability to bind water. It can be measured in different ways, e.g. as drip loss. When you heat the meat it is rather cooking loss you get. I can’t see that the method you describe is presented in the paper of Graham Trout. Why centrifuge at 10,000 g, when this is usually done at ordinary gravity (1 g) when it comes to drip loss?

*AU:* *Thank you for your attractive question. According to your comments, WHC has been removed from manuscript.*

**Results**

You have no values of feed intake or FCR for NG. Reformulate to levels of concentrate instead of feeding indoors.

*AU: Thank you for your suggestion and this sentence reformulate to levels of concentrate.*

Loin thickness did only differ between NG and CON, not between MC and HC. Please specify.

*AU:* *Thank you for your comment and your comment was done (Lines 272-273).*

RR is a part of the GIT, as you now have defined it in M&M, and should be presented as such also here.

*AU:* *Thank you for your comment. We decided to enter the performance parameters related to slaughter in the new table after blood parameters and behavior, and your suggestion done (Lines 302-304).*

It is enough of you present the result in whole minutes. Since you assumed that the behaviour continued for 5 min you don’t have that precision to actually measure in seconds.

*AU:* *Thank you for your constructive comment. Data were arranged in minutes and presented in bar plots (fig. 2) instead of as a table.*

…indicated that chemical composition of the meat did not differ…

*AU:* *Thank you for your comments, it has been changed accordingly.*

Delete “…in the CON fed lamb meat than that in NG lamb meat.”

*AU: Thank you for your offer and your offer has been made.*

Cooking loss, is it really in g/kg? WHC, what does the figures really mean? As said before, WHC is usually measured as drip loss so the latter would have been more proper to present in the table.

*AU:* *Thank you for your questions. The unit of cooking loss (%) was corrected.* *As I mentioned before, according to your comments, WHC has been removed from manuscript.*

**Discussion**

These two sentences say almost the same (ADG= weight gain).

*AU:* *Thank you for your comment and one sentence has been deleted because they were same.*

Again, RR is a part of GIT.

*AU: Thank you for your comment and it has been corrected.*

I assume you mean low fat cover for NG lambs? However, in your study you could not see a difference in back fat cover among the treatments. Or do you refer to another study? This is also valid for L 401-403; you own or someone else’s study?

*AU:* *Thank you for your comment. Back fat cover was not different among the treatments. I refer to another study and it has been corrected.*

Rephrase “…the consume energy and energy cost…”

*AU: Your comment has been considered and the sentence has been deleted.*

“Ruminating” instead of “rumination”.

*AU:* *Thanks for the comment and it has been changed.*

This sentence does not contribute to the text and can be omitted.

*AU:* *Your suggestions has been done and this sentence has been deleted.*

There was only one outdoor group*.*

*AU: Thank you and in response to your comment, there was only one outdoor group and the sentence was corrected.*

I think it is quite obvious that longer eating time is due to lower feed availability. Review if you really need the long reasoning above to come to that conclusion.

*AU: Your comment has been considered and the sentence has been deleted.*

But is longer eating time always a bad thing, is the welfare worse? Of course, if the animals are starving but their natural behavior is to seek for feed a large part of the day. I miss a discussion 2 “optimal” eating time, which automatically does not lead to a lower welfare due to longer eating time.

*AU: Thank you for your comment. Eating time is related to fattening system and feed availability. Based on feed availability, based on feed availability, Salem et al [23] found that the time spent grazing herbaceous vegetation actually corresponds to the time of searching vegetation residues above ground. Feed restriction may induce animals to increase their eating rate to maximize intake during periods of feed availability (Lines 362-365).*

*AU: Thank you for your suggestion and it has been changed.*

“…in the mixed group”, you need to explain what this means.

*AU: Thank you for your comment and was corrected.*

Rephrase “…, that aging improved…”.

*AU: Thank you for your comments. I deleted “…, that aging improved…” at the end of the sentence because it was not necessary and corrected this sentence (Lines 450-456).*

But did you have differences in chilling between the treatments? If so, this is confounding and should have been stated in the M&M. If you did not have different chilling, why do you think this could be the reason?

*AU: Thank you for your question. No, we haven’t differences in chilling between treatments. This sentence was deleted and used other reference.*

Pre-slaughter

*AU: Thank you and has been changed accordingly to your comment.*

Is 75 really the right reference? Earlier you referred to another paper when writing about WHC. And once again, WHC is a property of the raw meat and drip loss is used to describe it. Cooking loss is cooking loss and nothing else.

*AU: Thank you for your constructive comment and we have deleted this parameter to reflect the findings to your suggestions.*

**Conclusion**

To state that the NG lambs had lower welfare just due to longer eating time is doubtful. Then you need to put that in relation to what a “normal” eating time is. I think you should delete this statement.

*AU:* *Thank you for your comment and this statement has been deleted.*

You haven’t discussed profitability at all in the discussion. I agree that they probably are since they ate less concentrate but performed as well as HC. However, to state this I think you should mentioned this in the discussion as well.

*AU: I appreciate your comment. This was mentioned in this discussion. We found that lambs fed MC and HC had similar performance and carcass quality. It is debatable whether MC is an attractive option for finishing lambs or for maintaining lamb growth when concentrate prices are relatively high compared to lamb meat. On a commercial level, this could be beneficial in situations where concentrate prices are relatively high compared to lamb meat (Lines 424-428).*
