# Supplementary material for: Effects of nomadic grazing system and indoor concentrate feeding systems on performance, behavior, blood parameters, and meat quality of finishing lambs
Source: PLoS One. 2022 Dec 7;17(12):e0278669. doi: 10.1371/journal.pone.0278669 (PMC9728920; doi:10.1371/journal.pone.0278669)
Supplement: Attachment — Submitted filename: Response to Reviewers.docx [file pone.0278669.s006.docx]

Dear Dr. Antonio Humberto Hamad Minervino,

AU: We appreciate the opportunity to revise and resubmit this manuscript. Thank you for your constructive feedback on our original submission. We greatly appreciate the comments and criticisms. We revised the manuscript based on the advice and have addressed the reviewers' comments.

Sincerely,

Morteza H. Ghaffari

General comments

L 22-23 50:50% and 30:70%, make clear that it is on DM basis

*AU*: Thank you for your comment and your comment was used (*Lines 22-23)*.

L 28 You expected the CON-fed lambs to have better carcass yield so delete “interestingly”.

*AU*: Thank you for your comment and “interestingly” has been deleted.
L 35 You haven’t explained MC and HC, you can preferably do so in connection to 50:50 and 30:70 on lined 22-23.

*AU*: Thank you for your suggestion and this part has been moved to lined 22-23.
Materials and methods
L 101 It is unclear to me what you mean with “backyard”.
*AU*:Thank you for your comments. “backyard” has been deleted and I added “during each feeding, orts were collected and weighed before feed was given to the animals” (*Lines 101-102*).

Results
L 303 Weight is already included in EBW and HCW so you can delete “weight”.
*AU*: Thank you for your careful review and “weight” has been deleted (*Lines 304*).

L 303 and Table 4. I still think that RR should be included in GIT, not presented by itself since it is a part of the whole gastrointestinal tract. If you want to present it specifically you need to clearly state what you include in GIT (e.g. GIT excl RR).

*AU*: Thank you for your suggestion and RR parameter has been removed from manuscript.

Discussion
L 403 84%

*AU*: Thank you for your comment and it has been corrected (*Line 403*).
L 413 Think about how you want to present GIT and RR.

*AU*: Thank you for your suggestion. As I said before, I want to deleted RR parameter.

L 439 Glucose is one part in lowering pH after slaughter, but also glycogen has an important role. I think you can omit the part “… because of glucose level was not different.”

*AU*: Thank you for your comment and it has been deleted (*Line 438*).

Journal Requirements:

Please review your reference list to ensure that it is complete and correct. If you have cited papers that have been retracted, please include the rationale for doing so in the manuscript text, or remove these references and replace them with relevant current references. Any changes to the reference list should be mentioned in the rebuttal letter that accompanies your revised manuscript. If you need to cite a retracted article, indicate the article’s retracted status in the References list and also include a citation and full reference for the retraction notice.

AU: All references have been checked to ensure that they are correct and complete, and to avoid citing work that has been retracted.
