# Supplementary material for: Effects of nomadic grazing system and indoor concentrate feeding systems on performance, behavior, blood parameters, and meat quality of finishing lambs
Source: PLoS One. 2022 Dec 7;17(12):e0278669. doi: 10.1371/journal.pone.0278669 (PMC9728920; doi:10.1371/journal.pone.0278669)
Supplement: Attachment — Submitted filename: Response to Reviewers.docx [file pone.0278669.s007.docx]

SPECIFIC COMMENTS

Title:

•Please remove the nomadic term. In my humble opinion, it involves herd movement around a large geographic area. In this study a rotational grazing system was used probably in a small area (less than 1 km2).

*AU: Thank you for your comments* *and your explanation. In this study, nomadic grazing had a regular seasonal pattern of movement so if you allow the title of the article to be nomadic grazing.*

Abstract:

•Please remove paragraph related to profitability and sustainability. Neither production costs nor environmental impact were evaluated.

*AU: Your comment has been considered and remove paragraph related to profitability and sustainability.*

Introduction:

•There are many studies comparing grazing and intensive production systems based on TMR diets, and even the ratio of concentrate to forage on lamb performance and meat quality. The interest of this research has not been clearly described. What does this research contribute with respect to previous knowledge?

*AU: Thank you for your constructive comments. The requested paragraph has been added* “*Therefore, the appropriate production system and the weight of lambs before slaughter are of great importance in lamb production to obtain high quality lamb carcasses. However, the interest of this research was that feeding management plays an important role in feeding behavior, which leads to changes in plasma parameters and meat quality that are not well understood. This experiment will help provide more data and a better understanding of these relationships to support strategic feeding management to increase performance and improve carcass characteristics.”(Lines 57-62).*

Material & methods:

• What breed was used?

*AU: Thank you very much for your question and the study was carried in central Iran (Isfahan province, Iran) during the grazing season lasted 90 days (February 1, 2019 to May 1, 2019) using Turki-Ghashghaei breed sheep (Lines 75-76).*

• Were all animal’s male lambs?

*AU: Thanks for your question. Yes, all were male lambs.*

• Please indicate grazing area.

*Thank you for your attention and rotational grazing (RG, approximately 150 ha). The RG lambs were reared in Aghdash (31°36′09″ N 51°32′32″ E) in Vardasht Rural District of Semirom City (Isfahan Province, Iran) (Lines 86-88).*

• Was forage cut before mixing it with concentrate feeds?

*AU: Thank you for your careful review and your question. Yes, forage was cut before mixing it with concentrate feeds. The desired paragraph was added. As part of the harvesting process (Golchin Trasher Hay Co., Isfahan, Iran), alfalfa was chopped to a length of 30 mm by using a harvester with a screen size regulator. In the harvesting process, wheat straw was chopped to a theoretical cut length of 10 mm by using a harvester (Golchin Trasher Hay Co., Isfahan, Iran) (Lines 102-105).*

• Were orts daily collected or every week?

*AU: Thank you for your attractive question. During each feeding, orts were collected daily and weighed before feed was given to the animals, and the feed consumption was then measured (Lines 109-110).*

• What was the total area of the trough?

*AU: Thank you for your question. The desired paragraph was added. The total area of the trough was 30 cm (Lines 107-108).*

• Were all the animals able to access the feeder at the same time?

*AU: Thank you for your question. Yes, the desired paragraph was added. All the animals able to access the individual trough (30 cm) at the same time (Lines 107-108).*

*•* Do the authors think there might be differences between lambs in diet selection?

*AU: Thank you for your questions.* *Yes, we think there might be differences between lambs in diet selection and sorting index from CON-fed lambs* *were measured (data not shown).*

• Line 127. What does it mean (the NDF was expressed as ADF)?

*AU: Thank you for your question. It was removed from paragraph.*

• Line 212. Strange did not determine TBARs.

*AU: Thank you for your comment and changed by another reference (	Tarladgis BG, Watts BG, Younathan MT, Dugan JL. A distillation method for the quantitative determination of malonaldehyde in rancid foods. J. Am. Oil Chem. 1960; 37:44– 48).*

• Table 1. Ash content values seems to be too low [if you remove mineral content of additives (calcium carbonate, oxid manganese, salt, premix), an ash content value less than 0.5% can be estimated for the feed ingredients (98.5% of the total diet); it means that the mean value of organic matter content of the feed ingredients (alfalfa, straw, barley, …) would be greater than 99%].

*AU: The authors are grateful for your careful consideration. I apologize for the typo and the ash content has been corrected (Table 1).*

• Was behaviour assessed on only one day? Do the authors think that one day of behavioral recording is representative of the whole experimental period?

*AU: Thank you and to answer your question, it was very difficult to measure animal behavior under grazing conditions because the animals were grazing and their behavior was recorded every 5 minutes for 24 hours. In other sources, animal behavior was measured once for 24 hours during the whole period (Hamidreza Mirzaei-Alamouti et al., 2021).*

• Was LD dissected immediately after slaughter? Was not carcass refrigerated during 24 hours?

*AU: Thank you for your questions. Yes, the longissimus dorsi muscle was taken from the right half of the carcass and sampled for the following meat quality determinations immediately after slaughter. The color of a piece of meat (2.5 cm thick) from the thirteenth thoracic vertebra was determined 0 h post mortem (immediately after slaughter) and 24 h post mortem in the same section (lines 183-185). The color of meat depends on the concentration of a haeminic pigment, myoglobin, and its oxidation state (Renerre, 1990). Color measurements were taken immediately after slaughter and 24 hours postmortem to determine the effects of storage time (Sen et al., 2011). For this reason, we had to sample the other meat quality parameters at the same time and immediately after slaughter to ensure uniformity of the meat samples.*

•Meat samples for texture and TBARS analysis were frozen, but for drip loss determination they were storage under refrigerated conditions for 4 months (Lines 193-196) and reused. Is this interpretation correct? Does it make any sense to keep lamb meat for 4 months refrigerated at 4 º C?

*AU: We appreciate your constructive question. I'm sorry to confusing you. For drip losses and TBARS determination a sample of 80 and 100 g was placed from a plastic nylon bag stock in a refrigerated room (4ºC) for 24 h and other samples placed from freeze rome (-16 ºC) for 1, 2, 3 and 4 months (Lines 196-198).*

• Please modify description of meat chemical analysis.

*AU: Thank you for your constructive comment and the description was modify Meat quality and texture characteristics.*

•For instance: line 216. What does it mean? Was not the meat freeze-dried and then ground before the chemical analysis?

*AU: We highly appreciate important question. Meat chemical analysis samples were vacuum packed, frozen and stored at -20 °C until further analysis (AOAC, 2005). To measure fat, after dehydrating the meat using a freeze dryer were freeze‐dried, ground,* *and then it was measured (Lines 223-225).*

•Please remove intramuscular, as CP, fat and ash are intramuscular components in this case.

*AU: Thank you for your suggestion and intramuscular removed.*

• How were loin thickness and fat thickness measured?

*AU: Thank you for your question. An ultrasound scanner (Ultrascan 50, Linear 3.5 MHz Scan, Alliance Medical Inc) was used to measure the thickness of backfat and loin fat in live lambs between the third and fourth lumbar vertebrae (halfway between the last rib and the hip bone). (Lines 114-117).*

• Statistical analysis. Meat colour at 0 and 24 h postmortem was measured on the same slice. Therefore, a repeated measurement analysis should have been performed. In contrast, TBARS at 0,1, 2 and 3 months were determined on different slices, so a repeated measurement analysis would not be strictly necessary.

*AU: Thank you for your constructive comment* *and your comment done. Data with repeated measures (color of LD muscle) were analyzed with PROC MIXED from SAS (SAS 9.1; SAS Institute Inc; Cary, NC, USA) when variables were measured over time (Line 253).*

Results:

• Table 2. Please provide correct units: feed intake was g DM/day); FCR was g of DM/g ADG

*AU: Thank you very much for your comment. Feed intake was corrected as a kg DM/day. FCR was corrected as a kg of DM/kg ADG (Table 2).*

•It was not possible to compare the FCR of MC and HC because there is only one value per treatment;

*AU: Thank you for your comment. There is not only one value per treatments. I measured FCR for all lambs (CON- fed lambs: 20 numbers of data) and analysis by SAS (SAS 9.1; SAS Institute Inc; Cary, NC, USA).*

•Mean ADG value should not be very different from that estimated as the difference between the initial and final mean LBW divided by 90 day (for NG: (41.80-28.32/90 = 0.15 kg/day, but the mean value showed in table was 0.19).

*AU: Thank you for your questions. NO, ADG for all lamb calculated and put it 30 numbers of data for ADG to SAS (SAS 9.1; SAS Institute Inc; Cary, NC, USA) and data were extracted.*

• Table 4. i) Liver and GIT weights are not carcass characteristics;

*AU: The authors are very grateful for your comment. I removed Liver and GIT weights from carcass characteristics and I added* *Non-carcass parts for this parameters (Table 4).*

•Mean values should be checked. Slaughter weight estimated by dividing HCW by dressing percentage is much higher than the final LBW showed in table 2 (NG: 41.8 vs 43.4; MC: 46.5 vs 49.4; HC: 47.7 vs 50.6).

*AU: Thank you for your comment. Slaughter weight was not estimated by dividing HCW by dressing percentage. I changed this parameters as a Pre-slaughter BW, kg because were measured before being transferred to the slaughter route due to stress before slaughter (Table 2).*

Discussion:

•This section needs to be rewritten. It should be focused on results avoiding

*AU: Thank you for your constructive comments and I deleted some paragraph that data were not recorded.*

•Speculations. For example, feed intake, and rate of passage and particle size reduction were not recorded. NG lambs spent less time ruminating and it could be due to a lower NDF intake which could be due to a lower dry matter intake but also to a diet with lower NDF content (diet selection). Authors do not know the reason of the different behavior. These results should be interpreted with caution as they were collected for only 1 day and the experimental period lasted 90 days.

*AU: Your comment has been considered and the sentence have been deleted.*

•Mistakes. Starch intake does not increase rumination time (Line 371).

*AU: We appreciate your comment. This paragraph has been deleted.*

• Confusing paragraphs. For instance, lines 366-368. What does mean that feed restriction may induce animals to increase their eating rate?

*AU: Thank you for your comment and paragraph has been deleted.*

• Lines 368-369. What does mean to distinguish between rumen function and animal welfare?

*AU: The authors appreciate the delicate view of the reviewer. Rumination has been considered as a key component of digestion and intake by ruminants and as well as an important role in animal welfare (Krawczel and Grant, 2009). The natural behaviors that are most important to the health, welfare, and productivity of cows are resting, feeding, and rumination.*

•Line 422. What does “masculinization of rumen” mean?

*AU: Thank you for your question.* *The development of the rumen epithelium and muscularization are differently affected by the nature of the diet. Feed physical structure likely has the greatest influence on development of rumen masculinization and volume. Stimulation of rumen motility is governed by the same factors, particle size and effective fiber (Van Soest 1994).* *Feeding concentrate feeds in early life stimulates the development of the epithelium, while forages with large particle size or high fiber sources appear to be the primary stimulators of rumen masculinization and volume (Zitnan et al., 1998).*

• The use of inappropriate references. Line 435. Foti et al. evaluated the effect in suckling lambs, no in fattening lambs.

*AU: Thank you for your careful look. Foti et al. Was removed*

• Inadequate interpretation of the results. Lines 478-479. What are the data suggesting a high lipid oxidation in NG lambs?

*AU: Thanks for your comment. This sentence was deleted.*

Conclusions:

• Must be rewritten based on results. i) What parameters evaluating efficiency were determined?

*AU: Thank you for your question and this paragraph was removed.*

•what parameter were used to assec carcass quality? Only HCW?

*AU: Thank you for your question. The sentence was corrected based on your comment. The results of our study showed that lambs fed CON were higher pre-slaughter weight, HCW, and fat tail weight than lambs fed NG (Lines 479-480).*
